# Fabrication of Microgrooves by Synchronous Hybrid Laser and Shaped Tube Electrochemical Milling

**DOI:** 10.3390/ma14247714

**Published:** 2021-12-14

**Authors:** Yong Yang, Yufeng Wang, Yujie Gui, Wenwu Zhang

**Affiliations:** 1 Ningbo Institute of Materials Technology and Engineering, Chinese Academy of Sciences, Ningbo 315201, China; yangyong1994@nimte.ac.cn (Y.Y.); guiyujie@nimte.ac.cn (Y.G.); zhangww@nimte.ac.cn (W.Z.); 2 Zhejiang Province Key Laboratory of Aero Engine Extreme Manufacturing Technology, Ningbo 315201, China; 3 University of Chinese Academy of Sciences, Beijing 100049, China

**Keywords:** laser and shaped tube electrochemical milling, hybrid machining, microgroove, surface roughness, machining efficiency

## Abstract

The fabrication of deep microgrooves has become an issue that needs to be addressed with the introduction of difficult-to-cut materials and ever-increasing stringent quality requirements. However, both laser machining and electrochemical machining could not fulfill the requirements of high machining efficiency and precision with good surface quality. In this paper, laser and shaped tube electrochemical milling (Laser-STEM) were initially employed to fabricate microgrooves. The mechanisms of the Laser-STEM process were studied theoretically and experimentally. With the developed experimental setup, the influences of laser power and voltage on the width, depth and bottom surface roughness of the fabricated microgrooves were studied. Results have shown a laser power of less than 6 W could enhance the electrochemical machining rate without forming a deep kerf at the bottom during Laser-STEM. The machining accuracy or localization of electrochemicals could be improved with laser assistance, whilst the laser with a high-power density would deteriorate the surface roughness of the bottom machining area. Experimental results have proved that both the machining efficiency and the machining precision can be enhanced by synchronous laser-assisted STEM, compared with that of pure electrochemical milling. The machining side gap was decreased by 62.5% while using a laser power of 6 W in Laser-STEM. The laser-assistance effects were beneficial to reduce the surface roughness of the microgrooves machined by Laser-STEM, with the proper voltage. A laser power of 3 W was preferred to obtain the smallest surface roughness value. Additionally, the machining efficiency of layer-by-layer Laser-STEM can be improved utilizing a constant layer thickness (CLT) mode, while fabricating microgrooves with a high aspect ratio. Finally, microgrooves with a width of 1.79 mm, a depth of 6.49 mm and a surface roughness of 2.5 μm were successfully fabricated.

## 1. Introduction

Deep microgrooves, which have a width ranging from 0.7 mm to 2 mm and a depth of greater than 3 mm, are widely used in the critical components of various areas such as aerospace, medical, precision mold, and automotive manufacturing industries. Typically, microgrooves have found wide applications including the annular grooves of the aero-engine sealing parts, grooves of the turbine disc and the cooling channel of the rocket engine combustion chamber [1,2]. The machining accuracy and the surface roughness of microgrooves determine both the performance and life span of the equipment. With the introduction of various types of difficult-to-cut materials and ever-increasing stringent quality requirements, the fabrication of high-surface-quality microgrooves have posed a great challenge for the existing machining methods.

Mechanical machining methods have the defects of severe tool wear, poor chip removal and heat dissipation [3,4]. Non-traditional machining processes, such as electrical discharge machining (EDM), electrochemical machining (ECM), laser beam machining (LBM), and hybrid machining methods, have been increasingly employed to fabricate microgrooves. EDM removes material by a pulse spark discharge between electrode and workpiece, with high efficiency. Chu et al. has processed deep microgrooves utilizing the electrode jump motion in the EDM process and found that an electrode jump motion speed of 36 m/min could effectively improve the processing efficiency and accuracy [5]. Flaño et al. employed a thin foil electrode in an EDM process and showed that the 4 mm diameter holes in the electrode could reduce the processing time by 57% while machining microgrooves with a depth of 6.5 mm, and by 65% while machining 10 mm-depth microgrooves, respectively [6]. LBM could also process microgrooves with the translation of workpiece or laser beams in the lateral direction, with a high machining efficiency. However, both the EDM and the LBM process inherently remove materials by thermal effects, thus the processed microgrooves could always suffer from the recast layers and heat affected zone (HAZ) [7]. Besides, with the increase of machining depth of the LBM process, the surface quality and precision can deteriorate due to the difficulties in removing the debris and the shielding effects of the laser-induced plasma plume. ECM removes workpiece materials by the controllable anodic dissolution process, and the machining capacity is not limited by the mechanical properties of workpiece materials [8,9]. Furthermore, ECM could remove materials without tool wear, recast layers and heat-affected zone [10,11]. Hence, ECM possesses great potential in fabricating deep and narrow microgrooves with high surface quality. Zhang et al. proved that microgrooves with a width of 1.32 mm and a depth of 8.05 mm can be fabricated, utilizing the electrochemical milling with a shaped tube electrode [12]. However, the machining efficiency of ECM is lower than other unconventional machining methods, and the machining accuracy might be affected by stray current corrosion.

To enhance the machining efficiency and precision of ECM, the hybrid machining processes, which combine the ECM and other processes, have been increasingly proposed such as the electrochemical grinding process, hybrid laser and electrochemical process, and electric discharge and electrochemical process [13,14,15]. Hybrid laser and electrochemical machining (LECM) take advantage of the high efficiency of LBM and good surface quality of ECM [16]. Nowak et al. showed that laser-induced heating could accelerate electrochemical dissolution in the passivation and trans-passivation zone during the laser-induced wet chemical etching [17]. Long et al. utilized an excimer laser in the LECM process, and proved that LECM could overcome the recast layer, heat-affect zone and thermal stress inherent in LBM [18]. Tsao et al. revealed that laser assistance could accelerate the electrochemical etching rate and improve the process localization [19]. Desilva et al. proposed that the processing accuracy could be enhanced by 38%, and the materials removal rate (MRR) could be increased by 54% and 33% while machining aluminum alloy and stainless steel, during the laser-assisted jet electrochemical machining process [20]. However, to date, previous research was mainly focused on the materials removal mechanism of LECM, and the processability of microcavities of LECM. Few studies have considered the fabrication of deep and narrow microgrooves utilizing LECM.

Hybrid laser and shaped tube electrochemical machining (Laser-STEM) utilize a hybrid tubular electrode as both the cathode for ECM and an optical guide for the laser beam [21]. Laser-STEM combined the advantages of laser-material interaction, electrochemical machining, and laser-electrochemical interaction, which could make the material removal efficiency on the front machining gap continuously maintained at a high level [22]. Compared with the pure STEM process, Laser-STEM could promote the machining precision and MRR by 60.7% and 122.7% [23]. Laser-STEM processes have the capability of processing the large-depth small holes with a diameter of 1.25 mm and a depth of 5 mm on an aluminum alloy workpiece, free of a recast layer, which has been fabricated [24]. However, as a hybrid machining process, the current research on Laser-STEM was limited to the processability of the high-aspect-ratio small holes and explores the optimum processing parameters to improve the processing properties, such as machining efficiency, machining precision, and surface quality. The feasibility of Laser-STEM in processing microgrooves and three-dimensional microstructures has not been studied yet.

In this paper, the Laser-STEM process was introduced to the machining of deep and narrow microgrooves. Ni-based superalloys, which are widely used in hot-end components of aeroengines such as combustor and turbine arrangements, were utilized as a work material [25]. A three-dimensional hybrid laser and shaped tube electrochemical milling could be achieved by controlling the workpiece movement path, while taking the advantages of laser-electrochemical coupling effects, laser processing and localized electrochemical dissolution. Reaction products and heat could flow out of the machining zone immediately by the high-speed flow of electrolytes from the inner hole of the hybrid tubular electrode. The influences of laser power and voltage on the dimensions of the deep microgrooves, machining efficiency and surface roughness of the laser-assisted shaped tube electrochemical milling will be studied. Further, microgrooves with a large depth were fabricated using layer-by-layer Laser-STEM with the constant inter-electrode gap (CIEG) and constant layer thickness (CLT) mode.

## 2. Methods

### 2.1. Principles of Laser and Shaped Tube Electrochemical Milling

**Figure 1:**
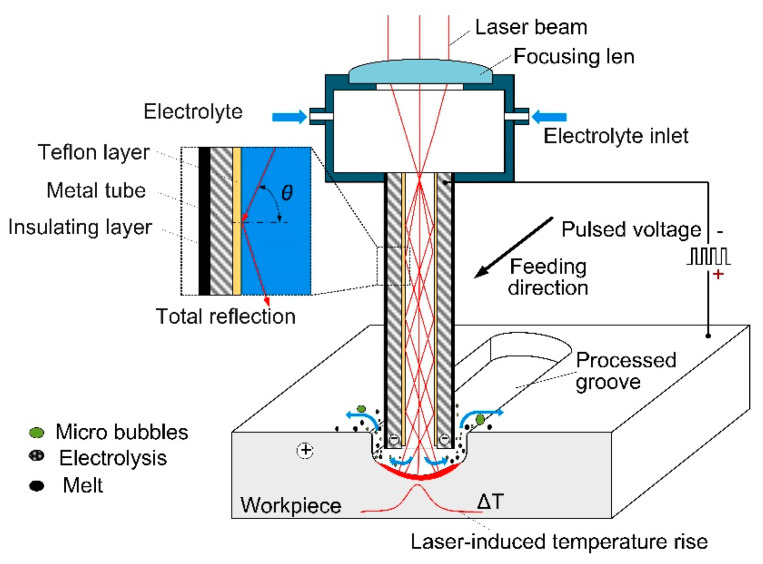
Mechanism of synchronous laser and shaped tube electrochemical milling (Laser-STEM).

Figure 1 shows the mechanism of the hybrid laser and shaped tube electrochemical milling (Laser-STEM), in which a tubular electrode was employed as both the tool cathode and optical waveguide. The innermost layer of the tubular electrode was a capillary tube with the optical refractivity lower than that of the electrolyte (*n* = 1.35), which served as a total reflective layer of the laser beam. A hollow metal capillary with an outer insulating coating on the external surface was adhered coaxially outside of the reflective layer and worked as the electrochemical machining electrode. The electrode could feed into the workpiece materials during the process similar to the tool electrode in the shaped tube electrochemical machining process, realizing the synchronous coupling of the laser beam and electrochemical reaction at the machining zone. The electrolyte flow was from the inside channel of the electrode into the machining gap; thus the reaction products and heat could flow out effectively.

**Figure 2:**
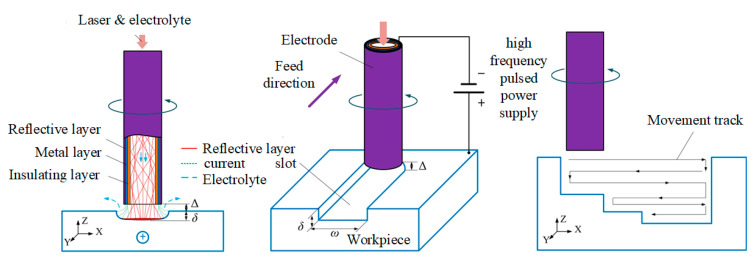
Schematic diagram of Laser-STEM for the fabrication of microgrooves and three-dimensional microstructures.

Figure 2 shows the schematic diagram of Laser-STEM for the fabrication of microgrooves and high-aspect-ratio microstructures. The electrode and workpiece were connected with the negative and positive polarities of the high frequency pulse power source, respectively. The initial interelectrode gap ∆ was pre-set between the end of the electrode and workpiece. Materials were removed under the synchronous coupling of the laser and electrochemical reaction. During the processing, the target machining structure was divided into several layers along the axis of the tubular electrode, the electrode could travel along the programmed path layer by layer.

### 2.2. Experimental Setup

**Figure 3:**
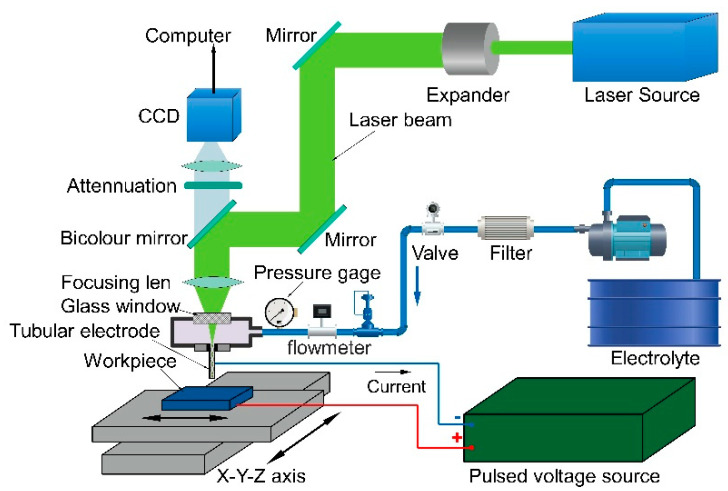
Experimental setup for Laser-STEM.

The schematic diagram of the experimental setup for the Laser-STEM was shown in Figure 3. A three-dimensional motion platform was utilized to achieve the precision control of the relative motion between the hybrid tubular electrode and the workpiece. The hybrid tubular tool electrode was clamped on the machining head and traveled along the *z*-axis. A gantry frame structure made of marble was adopted as the base. The linear displacement platform of the *x*-*y* axis was fixed on the marble platform, located below the *z*-axis. The workpiece and the electrolytic cell were fastened on the *x*-*y* axis platform so that the workpiece could move arbitrarily in the *x*-*y* plane. A laser source with a wavelength of 532 nm was employed, because the laser attenuation coefficient at this wavelength is smallest in water (4.5 × 10^−4^ cm^−1^), which could reduce the energy loss of the laser in the electrolyte and thus improve the energy utilization efficiency. The laser beam traveled by using the reflective mirrors and was focused on the entrance center of the hybrid tubular tool electrode. The position of the laser focal point could be observed by the charge coupled device (CCD) system, and the position of the electrode could be adjusted by a multi-freedom micro-displacement device. A high frequency pulse voltage was utilized as the energy source for the electrochemical dissolution. The electrolyte was filtered before flowing into the hybrid tubular tool electrode with a filtering accuracy of 1 μm.

## 3. Experimental Procedure and Materials

The effects of the parameters of the laser and high frequency pulse voltage on the performance of Laser-STEM were studied utilizing the developed experimental setup. The mechanism of the hybrid laser and electrochemical machining was studied under the different laser power density and pulsed voltage. Comparison experiments were also carried out with the shaped tube electrochemical milling (STEM). Inconel 718, with a thickness of 10 mm, was utilized as the workpiece, and the other experimental parameters are listed in Table 1. The electrode had an outer diameter of 1.2 mm and inner diameter of 0.5 mm, the retracted length of the electrode was set to 0.5 mm. The improvements of the retracted electrode on the Laser-STEM have been studied in the previous research [26].

The initial interelectrode gap (IEG) was set to 0.2 mm using a short-circuit between the end of the tubular electrode and the workpiece surface. The IEG was set by moving the electrode back while the two electrodes contacted physically. The moving speed of the hybrid tubular electrode was set to 1.8 mm/min, while the tool electrode moved along the programmed trajectory in a lateral direction. The workpiece was cleaned ultrasonically with ethylalcohol for 30 min after machining. A laser scanning confocal microscope was utilized to measure three-dimensional morphology and surface roughness of the machined microgrooves.

## 4. Results and Discussion

### 4.1. Effects of Laser Power on Laser-STEM

**Figure 4:**
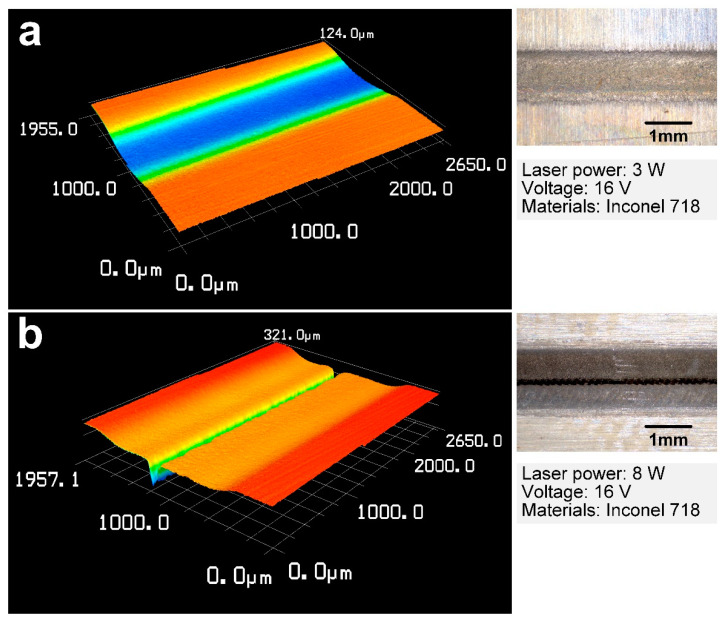
Three-dimensional morphology of the microgrooves machined by Laser-STEM at a laser power of (**a**) 3 W, and (**b**) 8 W.

Microgrooves were fabricated by laser and shaped tube electrochemical milling with the laser power increasing from 0 W to 10 W, with a single layer. Results showed that while the laser power exceeded a threshold value of 6 W, the central machining area can be directly removed by laser processing, resulting in a kerf along with an electrode moving path at the bottom of the processed microgroove. Figure 4 shows the three-dimensional morphology of the microgrooves machined at a laser power of 3 W and 8 W, respectively. A microgroove, with a relatively flat bottom, was obtained with a laser power of 3 W. At that laser power, the materials in the front machining gap could not be removed by laser processing directly. The distribution of the laser power density was similar to the Gaussian distribution. Thus, the materials removal rate in the central machining zone was much higher than that of the side of the microgrooves because of the uneven distribution of the laser power density in the machining zone. Microgrooves with a kerf on the center along the machining path were obtained with a laser power of 8 W, as shown in Figure 4b. To enhance the machining precision of the microgrooves, a laser power ranging from 0 to 6 W was used. Thus, in this study, the workpiece materials were removed by laser-assisted electrochemical machining. The laser-induced temperature rise could improve the electric conductivity of the electrolyte at the machining area and enhance the diffusion rate of the electrolytic products, contributing to a higher machining rate and localization, which are studied in the following experiments.

**Figure 5:**
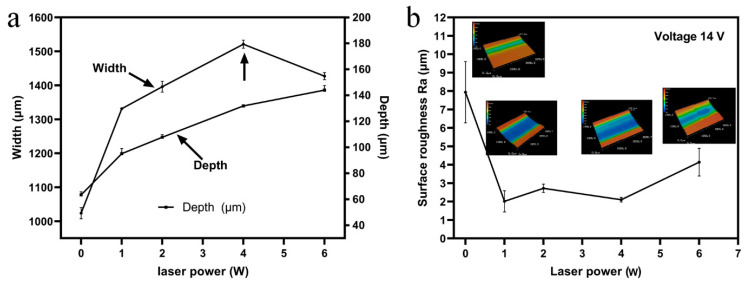
(**a**) Variation of the width and depth of the microgrooves fabricated by Laser-STEM with laser power. (**b**) Variation of the surface roughness Ra of the bottom of the microgrooves with laser power.

Figure 5 shows the variation of the width and depth of the machined microgrooves with the laser power. A laser power of 0 W to 6 W was applied to the machining area. Results showed that the depth of the machined grooves increased when the laser power increased from 0 W to 6 W. The width of the microgrooves increased when the laser power increased from 0 W to 4 W, and then decreased with a laser power of larger than 4 W. The materials removal rate of the electrochemical machining was directly related to the electric current density in the machining zone, based on Faraday’s law. The electric current density of the electrochemical dissolution can be expressed as [27]:

(1) $$i_{L}=i_{0}\exp\left[\frac{E_{0}\Delta T}{RT_{0}(T_{0}+\Delta T)}\right]$$

where *T*_0_ is the initial temperature of the processing zone, ∆*T* is the laser induced temperature rise, *i*_0_ is the electric current density of the ECM without laser-assist, *R* is the gas constant and *E*_a_ is the activation energy of the ECM. Hence, the laser-induced temperature rise could improve the electric current density for electrochemical machining. Additionally, the electric conductivity of electrolyte in the machining zone and the transport efficiency of particles would be enhanced with the increased temperature. Thus, the materials removal rate can be boosted with the increase of laser power, leading to an increase of the width and depth of fabricated microgrooves. While the laser power was larger than 4 W, as shown in Figure 5a, the localization of ECM was enhanced with the distribution of the current density tending towards the front machining gap with a machining depth increase.

**Figure 6:**
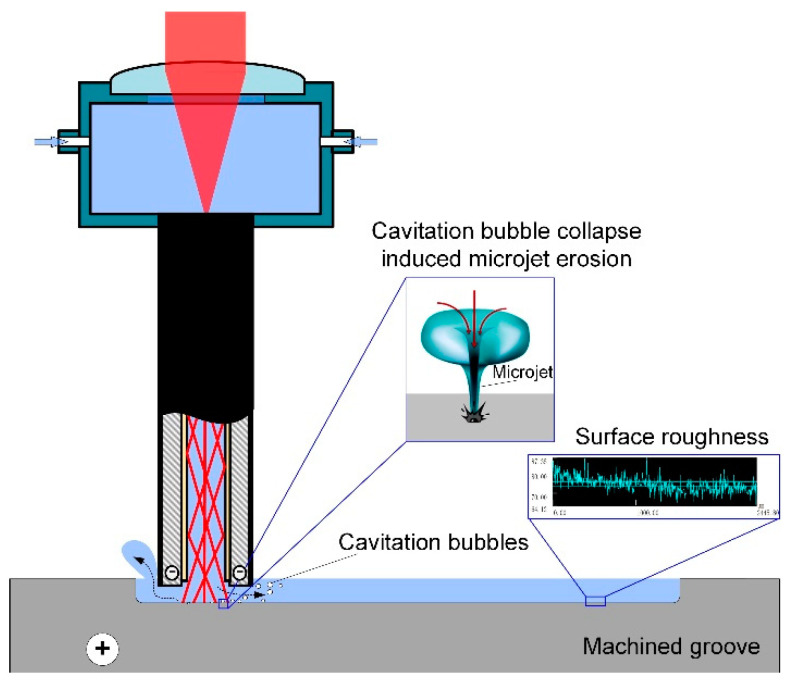
Schematic diagram of the influence of the micro-jet generated by laser-induced cavitation bubble collapse near the solid surface on the surface roughness.

Figure 5b shows the variation of surface roughness Ra at the bottom of the processed microgrooves with a laser power ranging from 0 to 6 W, and voltage 14 V. It was demonstrated that the surface roughness Ra decreased sharply when the laser power increased from 0 W to 1 W, which could be attributed to the positive effect of the machined surface smoothness with an increase of electric current density for electrochemical dissolution [28,29]. The surface roughness of the bottom surface of the laser-assist electrochemical machined microgrooves was kept constant when the laser power increased from 1 W to 4 W. Contrarily, the roughness Ra of the bottom surface increased while the laser power increased from 4 W to 6 W, due to an increased number of laser-induced cavitation bubbles in the machining zone. A micro-jet with high-pressure and high-speed was generated while the cavitation bubbles reached near the solid surface and collapsed [30], which would deteriorate the processed surface quality obtained by laser and shaped tube electrochemical milling, as illustrated in Figure 6.

During the Laser-STEM process, electrolyte flows out of the inner hole of the tubular electrode and fills the processing gap. The fluctuating effect of cavitation bubbles could produce a micro-stirring effect of the electrolyte, and the diffusion rate of reactive ions, electrolytic products, and reaction heat within the machining zone could be enhanced. Thus, the electric current density in the machining zone could be increased and the concentration polarization effect could be reduced. However, the cavitation bubbles collapsed, with the energy diminishing after multiple fluctuations; a micro-jet impact would act on the workpiece material nearby. The pressure on the surface of the workpiece under the micro-jet impact generated by the bubbles collapse can be expressed as [31]:

(2) $$P_{j}=\frac{\rho_{1}c_{1}\rho_{2}c_{2}}{\rho_{1}c_{1}{+\rho }_{2}c_{2}}v_{j}$$

where *ρ*_1_ and *ρ*_2_ are the density of the solution and workpiece, *c*_1_ and *c*_2_ are the propagation velocity of sound in the electrolyte and workpiece, and *v_j_* is the impact velocity of the micro-jet. It was suggested that the impingement pressure of the micro-jet could reach 400 to 500 MPa, and the micro-flow rate could exceed 100 m/s [32]. Hence, the machined surface quality would be affected by the erosion effect of the micro-jet impingement.

**Figure 7:**
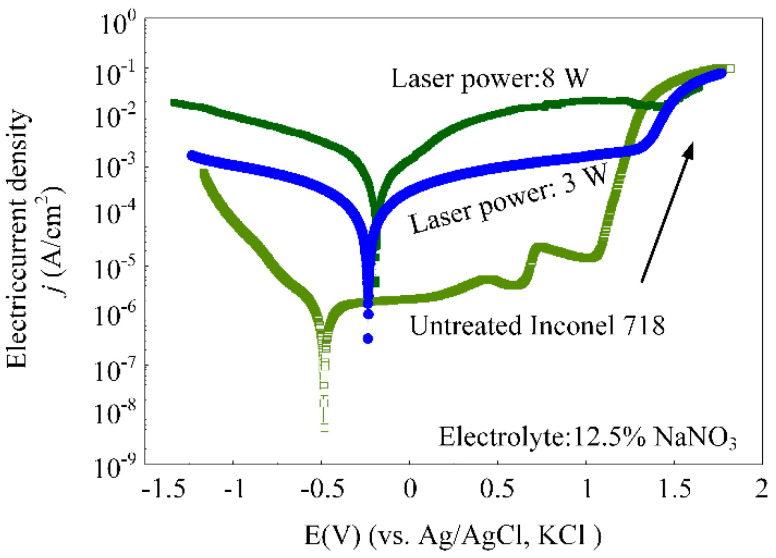
Electrochemical polarization curves of workpiece materials treated with different laser powers of 0 W, 3 W and 8 W.

The electrochemical polarization curves of workpiece materials were measured to study the effect of electrochemical machining characteristics while a laser of different powers was synchronized, coupling into the machining zone. As shown in Figure 7, the electric current density of the electrochemical machining increased with an increase of laser power, under the same voltage. Additionally, the over-passivation potential of the workpiece surface increased while the laser power increased, which could be attributed to a laser induced increase of the oxide layer thickness on the surface of the workpiece. Thus, the materials removal rate can be enhanced with an increase of laser power.

### 4.2. Effects of Voltage on Laser-STEM

**Figure 8:**
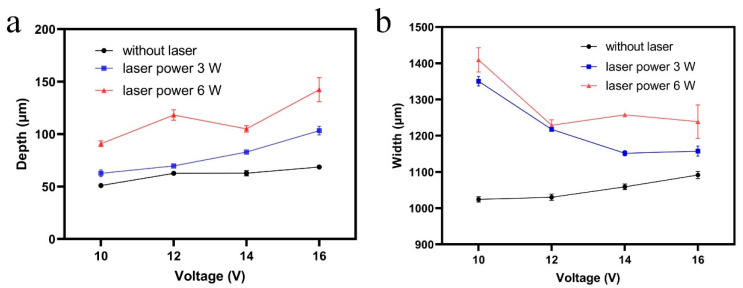
Variation of the (**a**) depth and (**b**) width of the microgrooves processed by Laser-STEM with voltage and laser power.

Voltage was the crucial factor of the material removal rate and machining precision in ECM. The voltage of 10 to 16 V was applied in Laser-STEM. Based on Ohm’s law, both the electric current density and material removal rate increased with an increase of voltage. Figure 8 shows the variation of the width and depth of the microgrooves with the voltage, demonstrating that the laser could effectively improve the material removal rate of the ECM process. As shown in Figure 8a, the depth of the microgrooves increased with the voltage, rising from 10 V to 16 V, and the laser power rose from 0 to 6 W, which could be attributed to the enhancement of the high depth machining capacity with the electric current density distribution on the end of the hybrid tubular electrode with the increase of voltage. However, Figure 8b shows a decreasing trend of the width of the microgrooves with the processing voltage while utilizing the synchronous laser assistance in the machining zone. The electric current distribution at the front machining gap increased when the laser was introduced to the ECM, and the machining capacity on the high depth increase. The machining side gap decreased by 62.5% while using a laser power of 6 W. Therefore, the synchronous laser assistance could enhance the machining localization of electrochemical dissolution.

**Figure 9:**
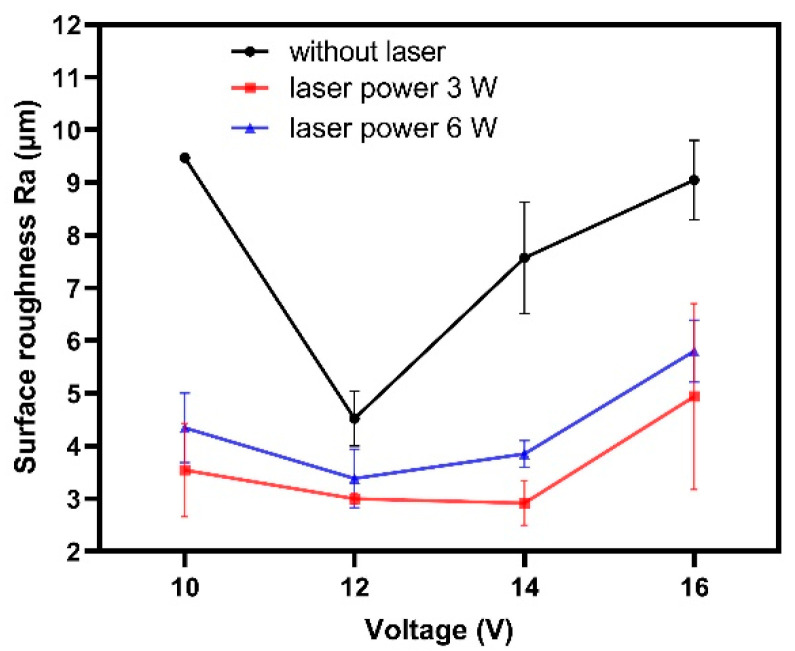
Variation of the surface roughness of the microgrooves processed by laser and shaped tube electrochemical milling with voltage and laser power.

Figure 9 shows the variation of the surface roughness Ra of the microgrooves processed by laser and shaped tube electrochemical milling with voltage and laser power. It was observed that the surface roughness Ra decreased when the voltage ranged from 10 V to 12 V, and then increased while the voltage ranged from 12 V to 16 V. The electric current density is positively associated with processing voltage, hence, the surface roughness decreased with an increase of current density while the voltage rose from 10 V to 12 V. However, the reaction speed of laser-assisted ECM was accelerated with the voltage increase. An uneven distribution of a large amount of reaction products was produced with a rising reaction speed, which would lead to the inhomogeneity of the electrical conductivity of the electrolyte and had an unfavorable impact on the uniformity of the material removal rate. Hence, uneven transport efficiency of electrochemical machining products on the front machining gap would affect the quality and roughness of the machined surface while the processing voltage ranged from 12 V to 16 V, as shown in Figure 9.

It was also found that the surface roughness Ra decreased when the laser power rose from 0 W. It could be attributed to the enhancement of the electrochemical reaction products diffusion efficiency on the reaction interface with the laser-induced local temperature rise. The surface roughness Ra of the machined surface decreased with the improvement of the uneven distribution of the electrochemical reaction products. The diffusion coefficient and diffusion rate of ECM products were closely related to temperature (*T*). The relationship between the diffusion coefficient (*D*) and the temperature (*T*) can be represented as [33]:

(3) $$D=D_{0}\exp(-\frac{Q_{d}}{RT})$$

where *D*_0_ is the pre-exponential factor independent of temperature, *Q*_*d*_ is the activation energy of diffusion, *R* is the gas constant, *T* is the absolute temperature. The diffusion coefficient of the ECM products increased with the temperature on the machining zone increase due to the rise of laser power. Hence, the laser-assistance effect was beneficial to reduce the surface roughness of the machined microgrooves, under certain processing voltage conditions. Further, results showed that the proper laser power of 3 W was preferred to obtain the smallest surface roughness value, that is, the smoothest machining surface.

### 4.3. Effect of Scanning Mode on Layer-by-Layer Laser-STEM

**Figure 10:**
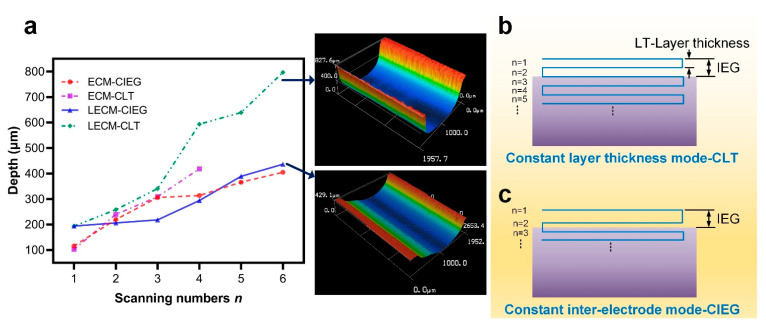
(**a**) Variation of the depth of the microgrooves processed by layer-by-layer Laser-STEM utilizing: (**b**) the constant layer thickness mode (CLT), and (**c**) the constant inter-electrode mode (CIEG).

To fabricate microgrooves or microstructures with a high aspect ratio, layer-by-layer Laser-STEM was utilized. Each layer thickness could be set as a constant distance (constant layer thickness mode, CLT) and a constant interelectrode gap mode (CIEG), as shown in Figure 10b,c. Comparative experiments were carried out to study the difference between the two proposed modes. Figure 10a shows the variation of the depth of the microgrooves processed by electrochemical machining (ECM) and laser-assisted shaped tube electrochemical machining (LECM) with the two modes, respectively. The voltage was set to 12 V and the laser power was set to 6 W. Results showed the depth of microgrooves increased with the increase of the scanning number *n*, while using the two modes, with and without laser assistance. It was shown that the depth of the microgrooves machined by the CLT mode was larger than the CIEG mode. The materials removal rate in each layer decreased with the increase of the machining depth of the microgrooves due to the difficulties in removing the electrolytic debris. Thus, the machining efficiency of the layer-by-layer laser and shaped tube electrochemical milling could be enhanced utilizing the CLT mode. However, the machining could not be continued in ECM while not using synchronous laser assistance when the scanning number exceeded 4; this is attributed to the electric short-circuits as the machining efficiency decreased at a large depth. In contrast, the machining depth could increase with the scanning number *n*, through the contribution of the enhanced machining efficiency of ECM assisted by the synchronous laser, as shown in Figure 10a. Besides, the depth of the microgrooves machined by ECM and LECM in CIEG mode was almost the same, which could be attributed to the limitation of the machining capability with a larger front interelectrode gap.

**Figure 11:**
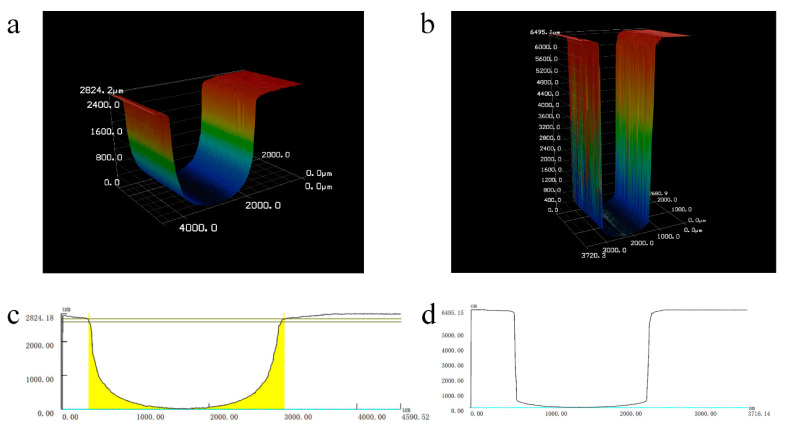
(**a**,**b**) Three-dimensional profiles of the deep and narrow microgrooves fabricated by laser and shaped tube electrochemical milling. (**c**,**d**) The cross-sectional profile of the correspondent microgrooves.

Figure 11 shows the three-dimensional profiles of the deep microgrooves machined by layer-by-layer laser and shaped tube electrochemical milling with a constant layer thickness of 0.1 mm, a voltage of 16 V and a laser power of 6 W. The depths of the microgrooves were 2.5 mm and 6.49 mm, and the widths were 1.93 mm and 1.79 mm, respectively.

## 5. Conclusions

In this paper, a laser and shaped tube electrochemical milling (Laser-STEM) process was proposed to fabricate deep and narrow microgrooves. Influences of the laser power and voltage on the depth, width and surface roughness of the microgrooves were studied, theoretically and experimentally. The conclusions can be summarized as follows:

1. When the laser power exceeded a threshold value of 6 W, a kerf processed by high power density was obtained at the bottom of the microgrooves. Microgrooves with a flat bottom were obtained with a laser power of smaller than 3 W, where the materials in the front machining gap were removed by laser-assisted electrochemical machining.
2. Results showed that the width of the microgrooves increased when the laser power increased from 0 W to 4 W, and then decreased with a laser power larger than 4 W. This contributed to increased machining efficiency of electrochemical machining due to the laser-induced temperature in the machining zone. The machining side gap decreased by 62.5%, while using a laser power of 6 W in laser and shaped tube electrochemical milling.
3. With a laser power of 0–4 W, the surface roughness was enhanced by the increased electric current density due to the laser-induced high temperature in the machining area. However, the surface roughness deteriorated, which was attributed to the intensified erosion effects of the micro-jet while the laser-induced cavitation bubbles collapsed.
4. The laser-assistance effects were beneficial to reduce the surface roughness of the microgrooves machined by Laser-STEM milling with the proper voltage. A laser power of 3 W was preferred to obtain the smallest surface roughness value.
5. The machining efficiency of layer-by-layer laser and shaped tube electrochemical milling can be enhanced utilizing the CLT mode while fabricating microgrooves with a high aspect ratio. Microgrooves with a width of 1.79 mm, a depth of 6.49 mm and a surface roughness of 2.5 μm were processed with a constant layer thickness of 0.1 mm, a voltage of 16 V, a feeding rate of the electrode of 1.8 mm/min and a laser power of 6 W.

Laser-STEM combines the advantages of laser processing and electrochemical dissolution, but the contradiction between the coupling efficiency of laser power and dimension of hybrid tubular electrodes has always been the bottleneck for the development of this process towards miniaturization, the coupling scheme needing further optimizing. The hybrid tubular electrode of a smaller dimension should be utilized in following studies. The future development of the Laser-STEM process will cover the miniaturization of the dimension of the hybrid tubular electrode and the fabricated structure. Additionally, the travel trajectory of the tubular electrode should be controlled and optimized to fabricate three-dimensional microstructures.

## Figures and Tables

**Table 1:** Experimental conditions for hybrid laser and shaped tube electrochemical milling.

Parameter	Value
Voltage (V)	10–16
Pulse frequency (KHz)	20
Duty cycle (%)	50
Electrolyte concentration (g/L)	12.5% NaNO_3_
Electrolyte pressure (MPa)	0.3
Electrolyte flow rate (mL/min)	100
Laser power (W)	1–6
Laser pulse width (ns)	16
Laser repetition frequency (KHz)	8
Wavelength (nm)	532
Temperature (°C)	24

## Data Availability

The datasets used or analyzed during the current study are available from the corresponding author on reasonable request.
